# Genome-Wide Detection of SPX Family and Profiling of *CoSPX-MFS3* in Regulating Low-Phosphate Stress in Tea-Oil *Camellia*

**DOI:** 10.3390/ijms241411552

**Published:** 2023-07-17

**Authors:** Juanjuan Chen, Xiaojiao Han, Linxiu Liu, Bingbing Yang, Renying Zhuo, Xiaohua Yao

**Affiliations:** 1 Key Laboratory of Tree Breeding of Zhejiang Province, Research Institute of Subtropical Forestry, Chinese Academy of Forestry, Hangzhou 311400, China; chenjuan@caf.ac.cn (J.C.); hanxj@caf.ac.cn (X.H.); lxliu9968@163.com (L.L.); bingbingyang1224@163.com (B.Y.); 2 Forestry Faculty, Nanjing Forestry University, Nanjing 210037, China

**Keywords:** SPX gene family, Tea-Oil *Camellia*, Pi stress, expression profiles, *CoSPX-MFS3*

## Abstract

*Camellia oleifera* a member of the family Theaceae, is a phosphorus (P) tolerator native to southern China. The SPX gene family critically regulates plant growth and development and maintains phosphate (Pi) homeostasis. However, the involvement of *SPX* genes in Pi signaling in Tea-Oil *Camellia* remains unknown. In this work, 20 *SPX* genes were identified and categorized into four subgroups. Conserved domains, motifs, gene structure, chromosomal location and gene duplication events were also investigated in the SPX gene family. Defense and stress responsiveness cis-elements were identified in the *SPX* gene promoters, which participated in low-Pi stress responses. Based on transcriptome data and qRT-PCR results, nine *CoSPX* genes had similar expression patterns and eight genes (except *CoPHO1H3*) were up-regulated at 30 days after exposure to low-Pi stress. *CoSPX-MFS3* was selected as a key candidate gene by WGCNA analysis. CoSPX-MFS3 was a tonoplast protein. Overexpression of *CoSPX-MFS3* in *Arabidopsis* promoted the accumulation of total P content and decreased the anthocyanin content. Overexpression of *CoSPX-MFS3* could enhance low-Pi tolerance by increased biomass and organic acid contents in transgenic *Arabidopsis* lines. Furthermore, the expression patterns of seven phosphate starvation genes were higher in transgenic *Arabidopsis* than those in the wild type. These results highlight novel physiological roles of the *SPX* family genes in *C. oleifera* under low-Pi stress, and lays the foundation for a deeper knowledge of the response mechanism of *C. oleifera* to low-Pi stress.

## 1. Introduction

Phosphorus (P) is among the necessary nutritional elements for the development and metabolism of all living organisms and plays an essential role in all life activities. As the second most important nutrient element, P is not only a constituent of important compounds (e.g., nucleic acids and proteins) in plants, but also regulates nearly all energy metabolism processes such as photosynthesis, respiration, energy conversion and enzymatic reactions [1,2,3]. Soil P is recognized as an integral part in Al, Fe or Ca salt. P can be taken up by plants in the form of orthophosphate (Pi, H_2_PO^4−^/HPO_4_^2−^) [4]. The concentration of available Pi is less than 10 µM in most soils [5]. Under different Pi environmental stresses, plants have evolved a sequence of adaptative mechanisms in morphological, physiological and molecular functions [6]. These mechanisms in plants are all controlled by complex and exquisite molecular regulatory networks, including an increasing number of transcription factors and Pi starvation responsive genes, microRNAs and other functional genes in the plant Pi signaling regulatory pathways.

The SPX domain (Pfam PF03105) was coined based on the first three letters of Suppressor of Yeast gpa1 (SYG1), Phosphatase 81 (PHO81) and Xenotropic and Polytropic Retrovirus receptor1 (XPR1) [7,8]. The protein-containing SPX domain is vital for the regulation of Pi signaling and Pi homeostasis. In yeast cells, *Pho84* is a high-affinity inorganic Pi sensor to maintain intracellular inorganic Pi uptake and transport [9]. As a low-affinity transporter, *Pho91* is in charge of the Pi allocation from vacuoles to cytosol [10]. The SPX protein domain is typically restricted to the N-termini of SPX gene family members. SPX domain proteins are assigned to four categories, namely, SPX, SPX-RING (SPX-Really Interesting New Gene), SPX-MFS (SPX-Major Facility Superfamily) and SPX-EXS (SPX-yeast ERD1, human XPR1 and yeast SYG1 protein subfamilies).

The SPX subfamily members contain only the SPX domain. In *Arabidopsis*, there are four SPX members, namely, *AtSPX1–4*. *AtSPX1* was distinctly upregulated by Pi starvation, and *AtSPX1* overexpression also increased the expression of phosphate starvation response (PSR) genes [11]. In potato (*Solanum tuberosum*), *StSPX2*/*3*/*5* are transcriptionally up-regulated under low-Pi stress [12]. The expression patterns of *ZmSPX4.1* and *ZmSPX4.2* were significantly different in maize (*Zea mays*) sensitive and insensitive to low-Pi condition [13].

The SPX-RING subfamily regulates Pi homeostasis under N limitation condition, and is commonly recognized as nitrogen limiting adaptation (*NLA*). The *NLA* gene can mediate the response of plants to nitrogen limitation, while *nla* mutant hyperaccumulates Pi, whose phenotype is similar to the *pho* mutant [14]. Meanwhile, loss of function of *NLA* enhances Pi accumulation by increasing several *PHT1s* at the protein level instead of the transcript level. *NLA* can direct the ubiquitination of *PHT1* in plasma membrane, by triggering clathrin-dependent endocytosis as well as transportation from endosome to vacuole [15].

The SPX-MFS subfamily contains the MFS domain. The MFS subfamily proteins contain 12–14 transmembrane domains and transport a broad range of substrates, including nucleotides, amino acids, ions and peptides [7]. In plants, the SPX-MFS family has been demonstrated to function as a Pi transporter [16,17,18]. *Arabidopsis* has three putative genes in this family (*At1g63010*, *At4g11810* and *At4g22990*). The SPX-MFS protein was also designated as a member of the phosphate transporter 5 family (*PHT5*) according to the systematic nomenclature of the *PHT1–PHT4* phosphate transporter protein [19,20]. In *Arabidopsis*, high expression of *PHT5* led to Pi accumulation, arrested growth and attenuated Pi influx into vacuoles [16].

The SPX-EXS subfamily consists of a hydrophobic region, namely the EXS domain (PF03124), and the PHO1 gene family members containing SPX and EXS domains are only found in eukaryotes [21]. There are 11 *Arabidopsis* (*AtPHO1*, *AtPHO1;H1–H10*) and 3 *Oryza sativa* (*OsPHO1;1*/*2*/*3*) members that are responsible for Pi homeostasis by transferring Pi from the root xylem to the shoot [22,23]. In *Arabidopsis*, *AtPHO1* overexpression led to a 2- to 3-fold rise in shoot Pi levels and significantly inhibited shoot development [24]. *OsPHO1;2* also regulated Pi transportation from rice plant roots to stems [25].

Tea-oil camellia (*Camellia oleifera*) is regarded as one of the four largest woody oil trees worldwide, along with coconut, olive and oil palm [26]. Tea-oil is obtained from the seeds, which contain various secondary metabolites and unsaturated fatty acids. It has been called “Oriental olive oil” due to its antioxidant activity and high oil content [27,28]. *C. oleifera* has been shown to have a high comprehensive utilization value. The fruit and seed shell of *C. oleifera* comprise a large amount of lignin, hemicellulose and cellulose, which have a wide range of industrial uses, and can be used to produce furfural, xylitol and activated carbon [29]. Tea-seed meal is rich in protein, polysaccharide and saponin and is used as raw material for light industry and chemical industry products [30]. *Camellia lanceoleosa*, a diploid wild species in the *Camellia* Sect. *Oleifera*, which is closely related to polyploid *C. oleifera* Abel, has gained increasing attention from scientific communities in recent years [31]. Although *SPX* family members have been diffusely studied in some crops and model plants, including rice [32], *Arabidopsis* [33], maize [13], wheat [34], rapeseed (*Brassica napus*) [35] and potato [36], a comprehensive analysis of *SPX* family members has not been performed in woody plants. Therefore, we initially explored the mechanism of *C. oleifera* response to reduced Pi stress in woody plants. Herein, all putative *SPX* genes in *C. lanceoleosa* genome were identified, and their structural characteristics and conserved motifs were analyzed. Moreover, the expression profiles of homologous *CoSPX* genes in the hexaploidy *C. oleifera* “Changlin166” roots under low-Pi stress were determined. The findings provide a reference basis for perspective functional experiments on the SPX gene family and a new theoretical framework for identifying novel genes, exploring genetic resources and improving Pi tolerance in tea-oil *Camellia*.

## 2. Results

### 2.1. Detection of C. lanceoleosa SPX Genes

The 20 SPX protein sequences of *Arabidopsis* were downloaded and utilized as query sequences. To determine putative SPX protein in *C. lanceoleosa*, the SPX (PF03105) domain was confirmed by Pfam, CDD and SMART (http://smart.embl-heidelberg.de/, accessed on 22 April 2023). There were 20 *SPX* genes (*ClSPXs*) identified in the genome of *C. lanceoleosa*, which were named based on the corresponding *Arabidopsis* homologs. Similar to wheat (*Triticum aestivum*), tomato (*Solanum lycopersicum*) and rice (*Oryza sativa*), the SPX subfamily contained the largest clade with 12 members, followed by 3 SPX-MFS, 3 SPX-EXS and 2 SPX-RING members (Table 1). The detailed information of all 20 SPX members, such as the number of gene ID, location, exons, protein properties, molecular weight, predicted isoelectric point values and estimated subcellular location, are presented in Supplemental Table S1. Further analysis uncovered that the ClSPX proteins consisted of 237–699 amino acids, carrying a molecular weight of 19.6–78.1 kDa. EXPASY analysis indicated that the SPX protein sequences had different isoelectric point (pI) values (range = 4.89–9.27). Subcellular location analysis showed that ClSPX proteins were localized in cell membrane, tonoplast, nucleus and chloroplast. This characteristic implies that different subfamilies may exhibit varying biology functions.

### 2.2. Phylogenetic Analysis of ClSPXs

**Figure 1:**
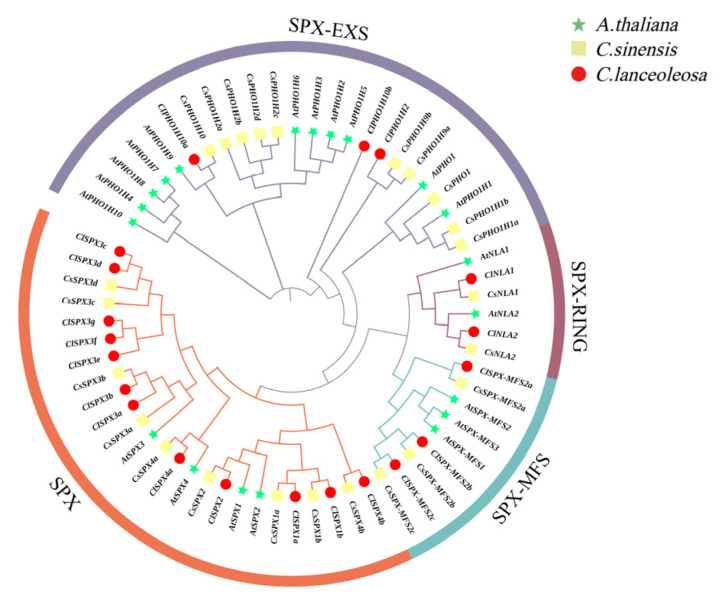
Phylogenetic relationships among the *SPX* genes from *C. lanceoleosa* (*Cl*), *C. sinensis* (*Cs*) and *Arabidopsis* (*At*). The phylogenetic tree of the three species was constructed according to the maximum-likelihood method with 1000 bootstrap replicates. Green stars represent *At*; yellow squares are *Cs*; red circles indicate *Cl*.

To illustrate the evolutionary relationship among SPX homologs in different plants, a phylogenetic tree was constructed comprising 20 ClSPXs in *C. lanceoleosa*, 20 AtSPXs in *Arabidopsis* and 24 CsSPXs in *C. sinensis*. The SPXs were clustered into four subclasses, namely, groups SPX, SPX-RING, SPX-MFS and SPX-EXS (Figure 1). The SPX group contained the most members, whereas the SPX-RING group contained the least, only two members.

### 2.3. ClSPX Protein Domain, Motif Composition and Gene Structure

**Figure 2:**
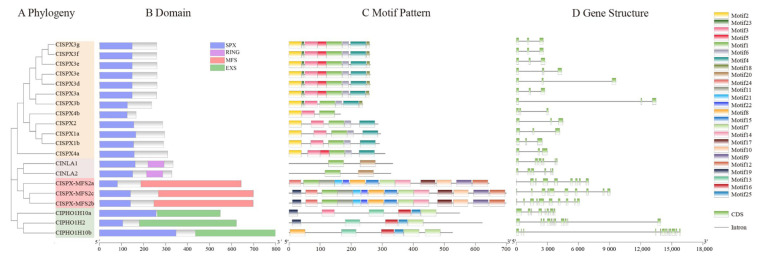
Phylogenetic relationships, domain, motif compositions and gene structures of *ClSPX* genes in *C. lanceoleosa*. (**A**) Phylogenetic analysis. (**B**) Domain analysis. (**C**) All conserved motifs in the ClSPX proteins were identified using the MEME program. Different motifs are highlighted with different colored boxes (numbered 1–25). (**D**) Gene structure. Exons are indicated by green, whereas gray lines represent introns.

The 20 ClSPX proteins were classified into four subfamilies according to the supplemental domains in protein structure, including SPX, SPX-RING, SPX-MFS and SPX-EXS (Figure 2B). To further examine the characteristics of *SPX* genes in *C. lanceoleosa*, 25 conserved motifs were identified by MEME analysis (Figure 2C). The specific information about the 25 putative motifs was listed in Supplementary Table S2. It was found that only the SPX subfamily contained motif 2. Motif 1 was specific to almost all ClSPXs (except SPX-EXS subfamilies), while motif 7 was only unique to the SPX-RING and SPX-MFS subfamilies (Figure 2C). Next, the structure of *ClSPX* genes was analyzed based on their exons and introns. Most *ClSPXs* exhibited similar exon–intron organization. ClSPXs with only the SPX domain have three exons, while ClSPX-RING, ClSPX-MFS2b/2c and ClSPX-MFS2a contain five, ten and nine exons, respectively (Figure 2D).

### 2.4. Assessment of Cis-Acting Elements in ClSPX Promoters

**Figure 3:**
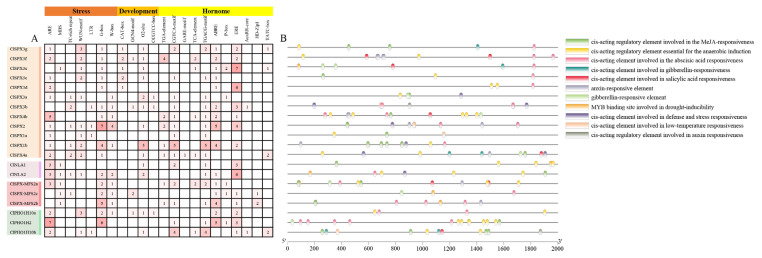
Analysis of cis-acting elements in the *ClSPX* promoter region. (**A**) The number of each cis-acting elements in the promoter region (2 kb upstream of the translation start site) of *ClSPX* genes. The depth of the color represents the number of cis-acting elements (**B**) Distribution of related cis-acting elements in *ClSPX* promoters.

The promoter regions of the upstream 2000 bp of 20 *ClSPXs* were assessed with PlantCARE. All cis-regulatory elements, such as TC-rich repeat, MBS, ARE, W-box, CAT-box, GCN4-motif, GARE-motif, ABRE, P-box, ERE and TATC-box elements, were assigned to three various categories: hormones, development and stress (Figure 3A). In addition, we identified 11 cis-regulatory elements, including defense and stress, MeJA stress response, auxin-responsive elements and others (Figure 3B), suggesting that *SPX* genes play vital roles in regulating plant abiotic stress [39,40].

### 2.5. Chromosomal Location and Synteny-Based Evaluation of ClSPX Genes

**Figure 4:**
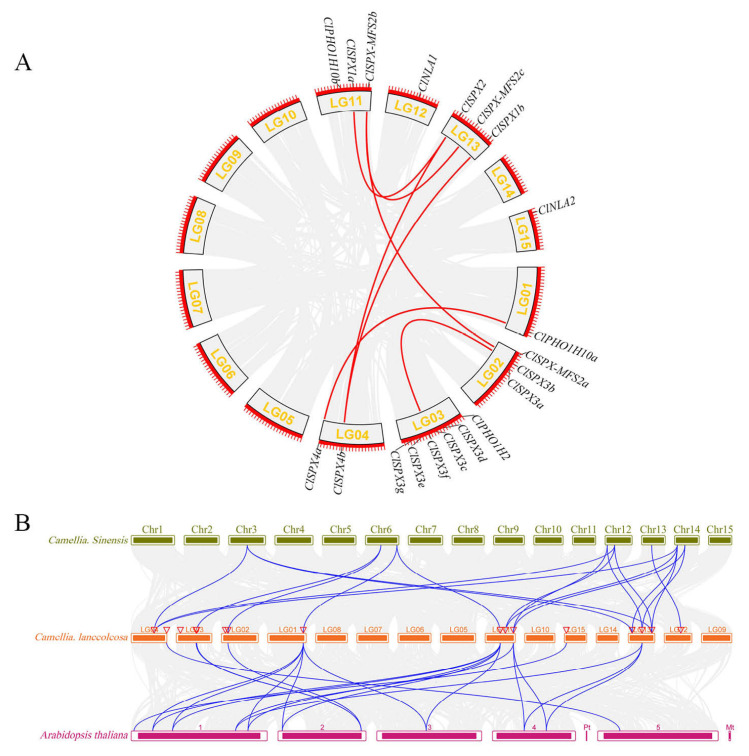
Synteny relationships. (**A**) Genome location and synteny of *SPX* genes in *C. lanceoleosa*. Gray lines indicate syntenic blocks in the *C. lanceoleosa* genome, while the red lines between chromosomes indicate segmentally duplicated gene pairs. (**B**) Synteny between *ClSPX* genes and genes in other species (*Arabidopsis* and *C. sinensis*). Number 1–5 means 5 chromosomes in *Arabidopsis*. Number LG01-LG15 means 15 chromosomes in *C. lanceoleosa*. Gray lines in the background represent collinear blocks in *C. lanceoleosa* and the other species, while blue lines indicate syntenic *SPX* gene pairs.

The physical locations of *SPX* genes were localized to the chromosomes (Chr) of *C. lanceoleosa* with TBtools [41]. The *ClSPX* genes were mapped to eight chromosomes, but were unevenly distributed. Non-uniform *SPX* gene distribution was observed on the chromosomes (Figure S1). Three *SPX* genes were found on chromosomes 2, 11 and 13, while six *SPX* genes were on chromosome 3. However, collinear genes were not present on the same chromosome. In fact, most genes possessed two similar genes on different chromosomes (e.g., *ClSPX-MFS2b*-*ClMFS-MFS2c/ClMFS*-*MFS2a* and *ClSPX4b*-*ClSPX2/ClSPX1b*), which formed two collinear gene pairs (Figure 4A). We found 7 segmental duplication gene pairs on six chromosomes. The Ka/Ks ratios of gene pairs were all <0.5, demonstrating that these genes may experience intense selection during the evolution process (Supplementary Table S3). Thus, assessing the evolutionary relationship of SPX family members may help to determine the functions of *ClSPX* genes. Syntenic maps of *C. sinensis* and *Arabidopsis* were constructed with *C. lanceoleosa* (Figure 4B). The results showed that 9 *ClSPX* genes exhibited syntenic relationships with 8 and 11 genes in *C. sinensis* and *Arabidopsis*, respectively (Supplementary Table S4). Moreover, 23 orthologous gene pairs were observed between *C. lanceoleosa* and *Arabidopsis*, and 21 orthologous gene pairs between *C. lanceoleosa* and *C. sinensis*. Six SPX collinear gene pairs were detected among *C. lanceoleosa*, *Arabidopsis* and *C. sinensis*. Most sites in these genes experienced intense purifying selection (Supplementary Table S4).

### 2.6. The Expression Profiles of CoSPX Genes in C. oleifera Tissues

As previously mentioned, *C. lanceoleosa* is closely related to polyploid *C. oleifera* Abel. At present, the main cultivar of *C. oleifera* is a polyploid plant. To further understand the *CoSPX* gene functions in polyploid *C. oleifera*, the expression profiles of all putative genes were analyzed in various tissues (Figure S2). The results demonstrated that most *CoSPX* genes have higher expression level in roots and stems. The same subfamily members of *CoPHO1H3*/*CoPHO1H5*, *CoSPX-MFS1*/*CoSPX-MFS2* and *CoNLA1*/*CoNLA2* had similar expression patterns.

### 2.7. The Expression Distribution of CoSPXs under Pi-Deficiency

**Figure 5:**
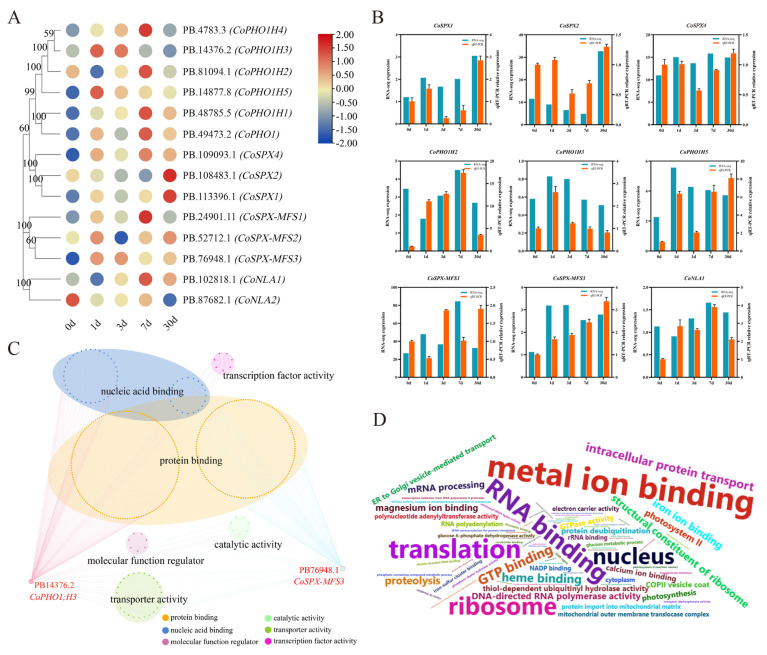
Expression profiles of *CoSPX* genes in plant roots under Pi stress conditions. (**A**) Gene expression data at 0, 1, 3, 7 and 30 days after the 5 µM Pi treatment were retrieved from an RNA-seq database. Expression levels are indicated by a gradient from low (blue) to high (red). (**B**) qRT-PCR validation and RNA-seq data of nine *CoSPX* genes. (**C**) *CoSPX-MFS3* gene co-expression regulatory network. The node genes are divided on the basis of the following six gene ontology (GO) terms, which are represented by different colors: protein binding, nucleic acid binding, molecular transducer activity, catalytic activity, transporter activity and transcription factor activity. (**D**) The frequency of GO terms.

The expression patterns of *CoSPX* genes were determined according to our previously reported RNA-seq data. Using the Fragments Per Kilobase of transcript sequence per Millions of base pairs sequenced (FPKM) values of our previous RNA-seq data, we found that almost all *SPX* genes could respond to low-Pi stress (Figure 5A). The results of transcriptome data and qRT–PCR (quantitative real-time polymerase chain reaction) showed that nine *CoSPX* genes had the same expression tendency, and eight genes (except *CoPHO1H3*) were upregulated at 30 days after exposure to low-Pi stress (Figure 5B).

### 2.8. Co-Expression Network of CoSPX Genes

To explore the roles of *CoSPX* genes in regulating Pi response gene expression, a co-expression modulatory axis was constructed according to the previous transcriptome analysis under low-Pi stress, among which two genes were identified as hub genes (Figure 5C). The hub gene-related nodes were involved in nucleic acid binding, protein binding, molecular function regulation, as well as transcription factor, catalytic, and transporter activities (Figure 5C). The frequency of GO terms was visualized using a word cloud (Figure 5D).

### 2.9. Sequence Alignment of ClSPX-MFS2b Homologous Genes

**Figure 6:**
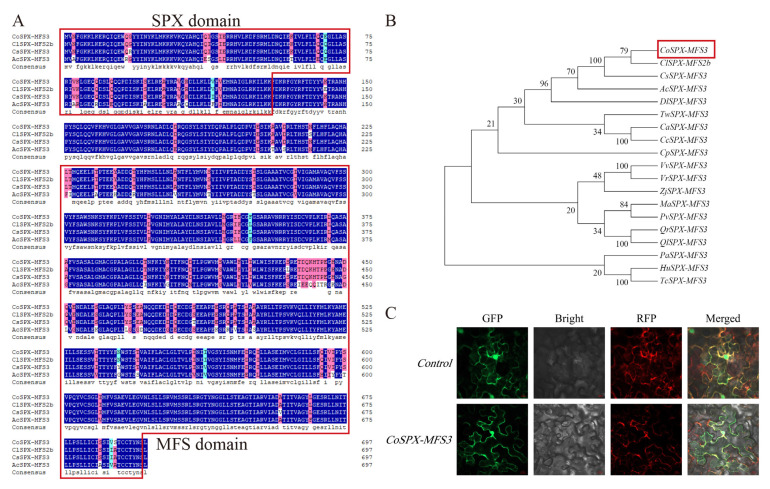
Identification of CoSPX-MFS3. (**A**) The amino acid sequence alignment from CoSPX-MFS3 and its orthologs. Red box means SPX domain and MFS domain, respectively (**B**) The phylogenetic relationships among the *SPX-MFS3* gene among 19 species. Red box means CoSPX-MFS3 (**C**) The subcellular localization of CoSPX-MFS3.

Based on our transcriptome data, a homologous gene *CoSPX-MFS3* in *C. oleifera* was selected as a key hub gene. Then, through multiple sequence alignment, we found the four closest species of CoSPX-MFS3 proteins with SPX and MFS domain (Figure 6A). By analyzing the SPX-MFS amino acid sequences of the 19 species, we found that CoSPX-MFS3 was the most closely related to ClSPX-MFS2b (Figure 6B). Thus, the functions of *CoSPX-MFS3* were analyzed by transcriptome combined with *C. lanceoleosa* diploid genome.

### 2.10. Localization of CoSPX-MFS3 in the Tonoplast

Subcellular localization indicated that CoSPX-MFS3-EGFP fusion proteins were gathered in the tonoplast (Figure 6C), which is in line with the prediction from Cell-PLoc.

### 2.11. Overexpression of CoSPX-MFS3 Enhances Pi Tolerance in Arabidopsis

**Figure 7:**
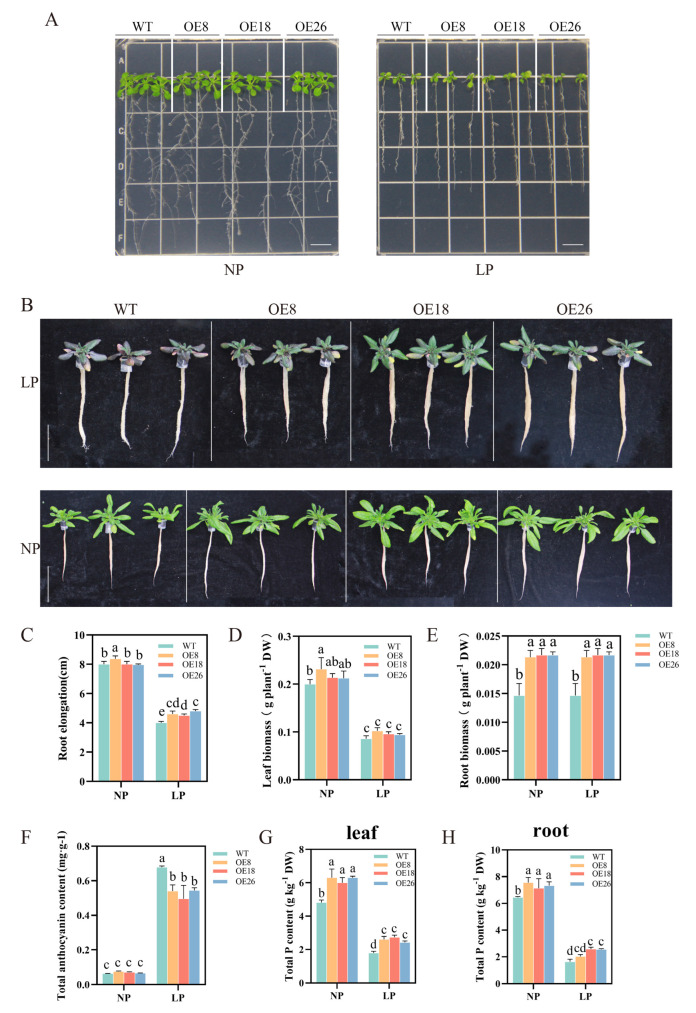
*CoSPX-MFS3*-overexpressing *Arabidopsis* plants exhibited strong tolerance to low-Pi stress. (**A**) Phenotypic analysis of *CoSPX-MFS3* overexpressing *Arabidopsis* under LP conditions. Seeds were germinated on 1/2MS agar medium for 5 d, then seedlings were transferred to NP (1 mM) and LP (5 μM) medium for 7 d. (**B**) Phenotypes of *CoSPX-MFS3* overexpressing lines and wild-type (WT) plants under NP and LP conditions. Bar = 5 cm. (**C**) Root elongation of seedlings Bar = 1.5 cm. (**D**,**E**) The biomass of roots and leaves. (**F**) The anthocyanin content of the WT and transgenic lines. (**G**,**H**) The total P content of leaves and roots. Bars represent the mean ± standard deviation (SD) of at least three independent biological replicates. Different letters above the bars represent significant differences at *p* < 0.05.

**Figure 8:**
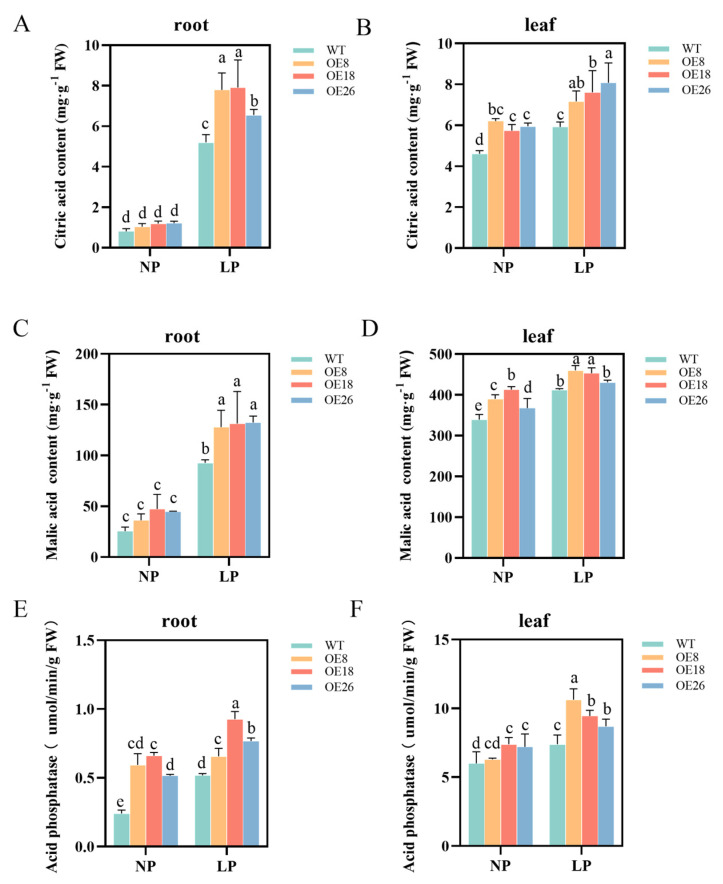
The organic acid content of *CoSPX-MFS3*-overexpressing *Arabidopsis* plants. (**A**,**B**) The citric acid content of the WT and transgenic lines. (**C**,**D**) The malic acid content. (**E**,**F**) The acid phosphatase content. Bars represent the mean ± standard deviation (SD) of at least three independent biological replicates. Different letters above the bars represent significant differences at *p* < 0.05.

**Figure 9:**
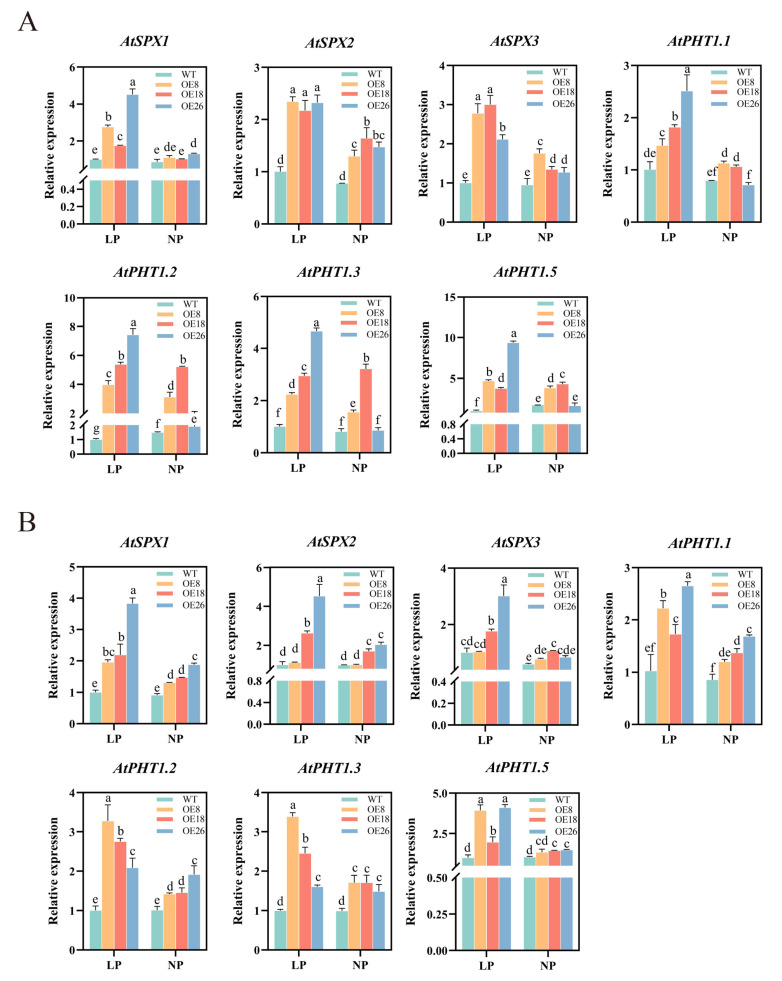
qRT-PCR analysis of the transcript expression of the homologous gene of WT and transgenic plants under two Pi levels in (**A**) root and (**B**) leaves. Different letters above the bars represent significant differences at *p* < 0.05.

To evaluate *CoSPX-MFS3* action during reduced Pi stress, *Arabidopsis* plants with *CoSPX-MFS3* overexpression (OE) were established. After qRT-PCR analysis of *CoSPX-MFS3* expression, the homozygous T3 lines were generated for further analysis (Figure S3). To examine potential *CoSPX-MFS3*-mediated regulation of Pi signaling and reduced Pi tolerance, we examined the phenotype of *Arabidopsis* plants overexpressing *CoSPX-MFS3* under Pi-deficiency (LP, 5 μM). The results showed a strong tolerance of *Arabidopsis* transgenic line with longer roots (Figure 7A,C). The accumulation of anthocyanin content is a marker of low-Pi stress in plants [42,43,44]. The degree of damage in leaves at 21 days after exposure to low-Pi stress was strongly elevated in wild-type (WT) versus *CoSPX-MFS3* OE lines (Figure 7B). The total anthocyanin concentration of transgenic plants was considerably diminished, compared to WT plants (Figure 7F). The root biomass was clearly distinct between WT and transgenic lines, regardless of low or normal Pi (NP, 1 mm) stress treatment (Figure 7D,E). The total P content in *CoSPX-MFS3* OE lines was increased (Figure 7G,H). Moreover, the malic and citric acid contents, as well as acid phosphatase activity were significantly higher in roots and leaves of the OE line, relative to WT plants (Figure 8A–F). All results demonstrated that *CoSPX-MFS3* could enhance Pi tolerance by increased biomass and organic acid contents. In addition, seven Pi-responsive homologous genes were selected for expression level verifications in transgenic *Arabidopsis*. The qRT-PCR results showed that all of them were up-regulated in transgenic *Arabidopsis* compared to WT (Figure 9). In summary, we conclude that *CoSPX-MFS3* may play an important role in mediating Pi tolerance in transgenic *Arabidopsis*.

## 3. Discussion

In response to low-Pi tolerance, plants form a series of biochemical and physiological adaptive mechanisms, which in turn enhance the absorption and utilization of soil Pi. Adaptive changes in plants under low-Pi stress are finely regulated by Pi signaling networks, in which *SPX* can regulate Pi-signaling networks in plants. Tea-oil *Camellia* mainly grows in acidic soil with very low P content in the southern area of China. Thus, assessing the biological functions of SPX proteins can elucidate the mechanisms underlying the adaption of *C. oleifera* to low-Pi pressure.

In our study, the total number of *SPX* genes in *C. lanceoleosa* was relatively similar to that in *Arabidopsis* (20) [11] and *Solanum lycopersicum* (19) [37], but less compared to wheat (46) [34], maize (33) [13] and *Brassica napus* (69) [35]. These differences in family members of different species may be due to environmental changes during evolution. The relationship between orthologs and paralogs shows that the diversity among the members of this gene family occurred before and after the divergences of monocots and dicots [45]. On the other hand, mutations in regulatory regions such as the promoter region and coding DNA regions probably caused the duplicated genes to have different expression patterns. [46,47]. Gene duplication is a crucial mechanism that can acquire new genes and create genetic novelty in organisms [48]. Eight putative *ClSPXs* were mapped to the corresponding chromosome according to the localization data of the *C. lanceoleosa* genome. The findings demonstrated that *ClSPXs* were non-uniformly distributed on the chromosomes of *C. lanceoleosa*. Meanwhile, it was found that segmental duplication occurred in *C. lanceoleosa* for expanding all *SPX* members during the process of evolution. The Ka/Ks rate is of great significance in reconstructing phylogeny and evolutionary selection [49]. Ka/Ks rates of <1, 1 and >1 represent purifying selection, neutral selection and positive selection, respectively [50]. The mean Ka/Ks value (0.177) of *C. lanceoleosa* demonstrated a strong purifying selection of *SPX* genes. In the same way, the Ka/Ks values of syntenic gene pairs between *C. lanceoleosa* and *Arabidopsis* or *C. sinensis* were less than 1. The Ka/Ks results imply that these paralogous gene pairs have experienced intense purifying selection during the evolutionary process.

The SPX conserved domain (SPX domain) plays a crucial role in modulating plant nutrient stress. In *Arabidopsis*, *AtSPX1* interacts with a unique four-stranded coiled-coil domain in *AtPHR1* [51]. In rice, SPX proteins (phosphate sensor) bind to OsPHR2 in high-phosphate condition, thus inhibiting OsPHR2 binding to P1BS elements in promoters of *OsRAM1*, *OsPT11*, *OsWRI5A* and *OsAMT3;1*, thus decreasing mycorrhizal-related gene expression and inhibiting mycorrhizal colonization [52]. In maize, *ZmSPX* genes (except *ZmSPX3*) were markedly induced by low-Pi stress [13]. In wheat, five *TaSPX* genes were up-regulated after Pi starvation treatment [34]. In this study, nine *CoSPX* genes had the same expression tendency, and eight genes (except *CoPHO1H3*) were up-regulated at 30 days after exposure to low-Pi stress, which was similar to other plants with low-Pi treatment. In general, SPX domain proteins as a low-Pi sensor. Two hub genes (*CoPHO1H3* and *CoSPX-MFS3*) were identified by WGCNA analysis. Meanwhile, co-expression analysis revealed that edge genes were involved in protein binding, nucleic acid binding, molecular function regulation, transporter activity, catalytic activity and transcription factor activity. These results demonstrate that SPX domain proteins serve an essential function in low-Pi stress perception and response.

In rice, *OsSPX-MFS3* was the first reported as a vacuolar Pi efflux transporter that modulates Pi homeostasis [18] and *OsSPX-MFS1* as a major Pi transporter that regulates leaf Pi homeostasis [38]. In *Brassica napus*, *BnA09PHT5;1b* and *BnCnPHT5;1b* were identified as two vacuolar Pi influx transporters [53]. The previous study revealed that SPX-MFS proteins primarily modulate Pi transport and homeostasis. Herein, we conducted a preliminary assessment of *CoSPX-MFS3* by heterologous transformed *Arabidopsis*. Overexpression of *CoSPX-MFS3* in *Arabidopsis* mainly enhanced low-Pi tolerance by increased biomass, organic acid content and total Pi content. Citric acid is an organic compound found in the roots of various plant species in response to Pi stress [54]. In white lupin, the citric acid content was increased at the later stage of Pi deficiency [55]. Although our results are different from rice and *Brassica napus*, this may be due to the long-term evolution of woody and herbaceous plants. Furthermore, overexpression of *CoSPX-MFS3* in *Arabidopsis* increased the transcription levels of a series of Pi starvation genes, including *PTs*, *SPX1*, *SPX2* and *SPX3*. This result is consistent with the previous findings that *AtSPX1* overexpression in *Arabidopsis* could increase the mRNA levels of *AtPAP2*, *AtACP5* and *AtRNS1* [11]. In summary, *CoSPX-MFS3* is a positive tolerator that responds to low-Pi stress. However, the detailed molecular mechanism of *CoSPX-MFS3* regulation in Pi acquisition and translocation need to be further identified.

## 4. Materials and Methods

### 4.1. Assessment of the SPX Family Genes in C. lanceoleosa

The *C. lanceoleosa* genome sequence and protein sequence information files were sourced from the NCBI database (https://www.ncbi.nlm.nih.gov/; accession number: PRJNA780224, accessed on 22 April 2023). To identify all SPX family members in *C. lanceoleosa*, the hidden Markov model (HMM) profiles of SPX domain (PF03105) were obtained using the Pfam database (http://pfam.xfam.org/, accessed on 22 April 2023). The 20 known SPX domain sequences from *Arabidopsis* were retrieved using the BLASTP search in TAIR (https://www.arabidopsis.org/, accessed on 22 April 2023) according to previous methods. After manually removing the redundant protein sequences of *C. lanceoleosa*, the candidate protein sequence containing a complete SPX domain (PF03105) was regarded as the final SPX protein sequence according to the NCBI Conserved Domain Database (CDD; https://www.ncbi.nlm.nih.gov/cdd/, accessed on 22 April 2023). These *C. lanceoleosa SPX* genes were renamed *ClSPXs*. The characteristics and subcellular localization of ClSPXs protein sequences were assessed using the ExPASY (http://Web.ExPASY.Org/protparam/, accessed on 22 April 2023) and Cell-PLoc 2.0 (http://www.csbio.sjtu.edu.cn/bioinf/Cell-PLoc/, accessed on 22 April 2023) tools, respectively.

### 4.2. Construction of Phylogenetic Trees

Full-length protein sequences from *Arabidopsis* and *Camellia sinensis* were acquired from the NCBI protein database to determine their evolution. The definitized amino acid sequences of ClSPXs were subjected to multiple sequence alignment via ClustalW (https://www.genome.jp/tools-bin/clustalw, accessed on 22 April 2023). The maximum-likelihood criteria in MEGA 7.0 were used to establish phylogenetic trees, with Poisson corrections and 1000 bootstrap replicates. The recognized *ClSPX* genes were assigned to distinct categories based on the *AtSPXs* stratification. Interactive Tree of Life (iTOL.7) software (https://itol.embl.de/, accessed on 22 April 2023) was employed for visualization and modification of the phylogenetic tress.

### 4.3. Assessment of ClSPX Gene Domain, Structure and Motif Sequences

The intron/exon map structure was drawn using the tools of the Gene Structure Display Server v2.0 (http://gsds.gao-lab.org/, accessed on 22 April 2023) platform. The conserved motifs in ClSPX proteins were determined using the MEME program v5.0.5 (http://meme-suite.org/tools/meme, accessed on 22 April 2023), with a maximum of 25 motifs and optimal motif width range of 6–50 amino acid residues.

### 4.4. Determination of Cis-Acting Elements in ClSPX Promoter

The 2000-bp sequences upstream of the *ClSPX* translation initiation codon were analyzed through the PlantCARE database in order to determine the cis-regulatory elements.

### 4.5. Chromosomal Location and Synteny-Based Evaluation of ClSPX Genes

The chromosomal location data of *ClSPXs* were extracted from the *C. lanceoleosa* genome annotation file. This information was also used to construct chromosomal mapping by TBtools [41]. Gene duplication assessment was conducted via the One-Step MCScanX function in TBtools, and the result was visualized by the CGview tool [41].

### 4.6. CoSPXs Expression Profiles

To determine the expression profiles of *CoSPX* at different stages (0, 1, 3, 7 and 30 days), the FPKM values of *CoSPX* genes at five treatment stages of *C. oleifera* were obtained from our previous RNA-seq data [56]. The expression profiles of *CoSPXs* at different stress stages were analyzed based on their FPKM values, and a heatmap was produced using the R heatmap function (gplots). Z-score normalization was used to normalize the expression values. Total RNA isolation was employed by the RNA kit (Aidlab Biotechnologies Co., Ltd., Beijing, China) following the manufacturer’s instructions. To conduct reverse transcription, PrimeScriptTM RT Master Mix (TaKaRa, Dalian, China) was used. Then, quantitative real-time PCR (qRT-PCR) analysis was conducted on the 7300 Real-Time PCR System (Applied Biosystems, Shanghai, CA, USA) with the help of the TB Green^®^ Premix Ex Taq™ II (Tli RNaseH Plus, Takara). The “Genes” module in SPDE software (Version 2.0)was used to design gene-specific primers [57]. The *GAPDH* gene served as the endogenous control for *CoSPXs* [58]. Actin gene was chosen as an internal reference for AtSPXs. Relative gene expression was computed via the 2^−ΔΔCt^ formula [59]. The employed primers are detailed in Supplemental Table S5.

### 4.7. Co-Expression Regulatory Network of CoSPX Genes

Among all co-expressed genes, *CoSPX* genes exhibited the largest interconnection. Thus, they were established as hub genes. Edge genes were annotated by GO terms, and their frequency was visualized in a cloud word (https://www.bioladder.cn/web/#/chart/20, accessed on 22 April 2023). Finally, the co-expression regulatory network was performed in Cytoscape 3.8.2.

### 4.8. Multiple Sequence Alignment, Subcellular Localization of CoSPX-MFS3

The SPX-MFS3 amino acid sequences of 19 different species were downloaded from NCBI for evolutionary analysis, and a multiple sequence alignment was constructed. The CoSPX-MFS3 coding sequences were fused with the mGFP-encoding sequences in the pMDC43 expression vector with the CloneExpress II One Step Cloning Kit (Vazyme, Nanjing, China). For transient expression analysis, the recombinant plasmids were incorporated into *Agrobacterium tumefaciens* EHA105 cells, prior to transfer to *Nicotiana benthamiana* leaves. The empty vectors were employed as controls. After three days, the transient expression of GFP-fusion proteins was determined using an LSM900 confocal microscope imaging system (Zeiss, Shanghai, China). mCherry-labeled vacuole markers were used to visualize the tonoplast.

### 4.9. Ectopic CoSPX-MFS3 Expression in Arabidopsis and Pi Exposure

The full-length CDS of *CoSPX-MFS3* was amplified from *C. oleifera* and inserted into the *CaMV 35S* promoter-regulated binary vector pEXT06 employing the ClonExpress II One Step Cloning Kit (Vazyme). Subsequently, *A. tumefaciens* GV3101-regulated transformation of *Arabidopsis* was employed. The transgenic *Arabidopsis* seeds were harvested from individual plants, and positive lines were identified on 1/2 MS medium using hygromycin until homozygous transgenic *Arabidopsis* lines were obtained. Using 75% ethanol, the homozygous seeds of *Arabidopsis* underwent surface sterilization three times, prior to a 24-h incubation at 4 °C. After incubation, the seeds were placed on petri dishes containing Pi-sufficient (normal Pi [NP], 1 mm Pi) medium for 5 days, prior to transfer to Pi-deficient (low Pi [LP], 5 μM Pi) medium for 7 days with a 16:8-h light/dark cycle at 22 °C. Wild-type (WT) plants and T3 homozygous transgenic lines were cultivated in 1/2-strength Hoagland solution (Coolaber, China) for one week. Subsequently, the plants were placed in 1/2-strength phosphorus-free solution and grown for three weeks. The fresh roots and leaves were immediately frozen in liquid nitrogen and kept at −80 °C. The roots and leaves were harvested for the determination of biomass.

### 4.10. Measurement of Total Pi Content

For determination total P content, the method described in a previous study [60] were strictly followed.

### 4.11. Determination of Anthocyanin and Organic acid Content

Fresh roots and leaves (0.1 g) of *Arabidopsis* were crushed in liquid nitrogen and incubated with 1% HCl-methanol for 24 h. The optical density values of anthocyanin were recorded at 530 nm (Tang et al., 2022). Commercial kits (Geruisi-bio, Suzhou, China) were employed for the detection of malic acid (Lot. G0862W48), citric acid (Lot. G0864F) and acid phosphatase (Lot.G0903W) contents. Briefly, *Arabidopsis* samples (0.1 g) were extracted using the kit’s buffer, followed by centrifugation (10,000 rpm, 15 min, 4 °C) [41]. The assays were performed in triplicate.

## 5. Conclusions

In summary, 20 *SPX* genes were identified in the *C. lanceoleosa* genome, and their conserved domain, motif distribution, gene duplication, cis-acting elements and chromosomal distribution were analyzed. Segmental duplication event was the key factor affecting the evolutionary process of the SPX gene family in *C. lanceoleosa* based on collinearity analysis. Additionally, the expression distribution of *SPX* genes in low-Pi stress conditions were analyzed using transcriptome data and qRT-PCR experiment. A hub gene *CoSPX-MFS3* induced by low-Pi stress could enhance low-Pi tolerance in transgenic *Arabidopsis*. Altogether, these findings present a reference basis for the enhanced understanding of the physiological roles of *SPX* genes in *C. oleifera*.

## Figures and Tables

**Table 1:** Number of *SPX* genes in seven plant species.

Species	SPX	SPX-EXS	SPX-MFS	SPX-RING	Total	Reference
*Arabidopsis*	4	11	3	2	20	[11]
Wheat	15	12	12	7	46	[34]
Maize	7	15	9	2	33	[13]
*Brassica napus*	11	43	8	7	69	[35]
*Solanum lycopersicum*	7	6	4	2	19	[37]
Rice	6	3	4	2	15	[38]
*C. lanceoleosa*	12	3	3	2	20	This study

## Data Availability

The *C. lanceoleosa* genome sequence and protein sequence information files were sourced from the NCBI database (accession number: PRJNA780224).

## Supplementary Materials

The following supporting information can be downloaded at: https://www.mdpi.com/article/10.3390/ijms241411552/s1.

## Abbreviations

P	Phosphours
Pi	Phosphate
LP	Low Pi
NP	Normal Pi
PSR	Phosphate starvation respsonse
SYG1	Suppressor of Yeast gpa1
XPR1	Xenotropic and Polytropic Retrovirus receptor1
NLA	Nitrogen Limiting Adaptation
ABRE	Abscisic acid response element
PHT5	Phosphate transporter 5 family
FPKM	Fragments Per Kilobase of transcript sequence per Millions base pairs
NJ	Neighbor-joining
HMM	Hidden Markov Model
qRT-PCR	Quantitative real-time polymerase chain reaction
