# Supplementary material for: Genome-Wide Detection of SPX Family and Profiling of CoSPX-MFS3 in Regulating Low-Phosphate Stress in Tea-Oil Camellia
Source: Int J Mol Sci. 2023 Jul 17;24(14):11552. doi: 10.3390/ijms241411552 (PMC10380294; doi:10.3390/ijms241411552)
Supplement: Supplementary file 1 [file ijms-24-11552-s001.zip › Table S1.pdf]

Table S1 The basic information of *ClSPX* gene families

Gene name	Gene ID	Location	Exon	Protein length (aa)	MW (Da)	pI	Subcellular location
ClPHO1H10a	C.lanceoleosa_LG01_G03739.t1	LG01	8	549	64082.76	9.27	Cell membrane
ClSPX2	C.lanceoleosa_LG13_G00254.t1	LG13	3	287	32798.72	5.46	Nucleus
ClSPX1a	C.lanceoleosa_LG11_G01509.t1	LG11	3	295	33562.58	5.72	Nucleus
ClSPX3a	C.lanceoleosa_LG02_G00684.t1	LG02	3	259	30145.78	6.26	Nucleus
ClSPX4b	C.lanceoleosa_LG04_G02574.t1	LG04	3	166	19625.19	5.88	Nucleus
ClSPX3g	C.lanceoleosa_LG03_G02574.t1	LG03	3	260	30294.02	5.81	Tonoplast
ClSPX4a	C.lanceoleosa_LG04_G03850.t1	LG04	3	309	34972.45	4.89	Nucleus
ClSPX3e	C.lanceoleosa_LG03_G02564.t1	LG03	3	261	30319.84	5.55	Tonoplast
ClSPX3f	C.lanceoleosa_LG03_G02558.t1	LG03	3	260	30234.96	5.81	Tonoplast
ClSPX3c	C.lanceoleosa_LG03_G02550.t1	LG03	3	261	30138.72	5.74	Tonoplast
ClSPX3d	C.lanceoleosa_LG03_G02542.t1	LG03	3	261	30291.97	6.06	Chloroplast
ClSPX3b	C.lanceoleosa_LG02_G00681.t1	LG02	3	237	27508.8	8.94	Nucleus
ClSPX1b	C.lanceoleosa_LG13_G02593.t1	LG13	3	291	33563.69	5.18	Tonoplast
ClPHO1H2	C.lanceoleosa_LG03_G00703.t1	LG03	11	622	71315.95	9.61	Cell membrane
ClSPX-MFS2c	C.lanceoleosa_LG13_G01292.t1	LG13	10	699	77987.42	6.18	Tonoplast
ClSPX-MFS2b	C.lanceoleosa_LG11_G02584.t1	LG11	10	698	78063.44	6.00	Tonoplast
ClNLA1	C.lanceoleosa_LG12_G02084.t1	LG12	6	334	38626.71	7.47	Nucleus
ClSPX-MFS2a	C.lanceoleosa_LG02_G00476.t1	LG02	9	644	71841.94	4.85	Cell membrane
ClPHO1H10b	C.lanceoleosa_LG11_G01096.t1	LG11	12	526	61804.02	8.85	Cell membrane
ClNLA2	C.lanceoleosa_LG15_G00224.t1	LG15	6	328	37724.12	8.56	Nucleus
