# Supplementary figures and images for: Genome-Wide Detection of SPX Family and Profiling of CoSPX-MFS3 in Regulating Low-Phosphate Stress in Tea-Oil Camellia
Source: Int J Mol Sci. 2023 Jul 17;24(14):11552. doi: 10.3390/ijms241411552 (PMC10380294; doi:10.3390/ijms241411552)

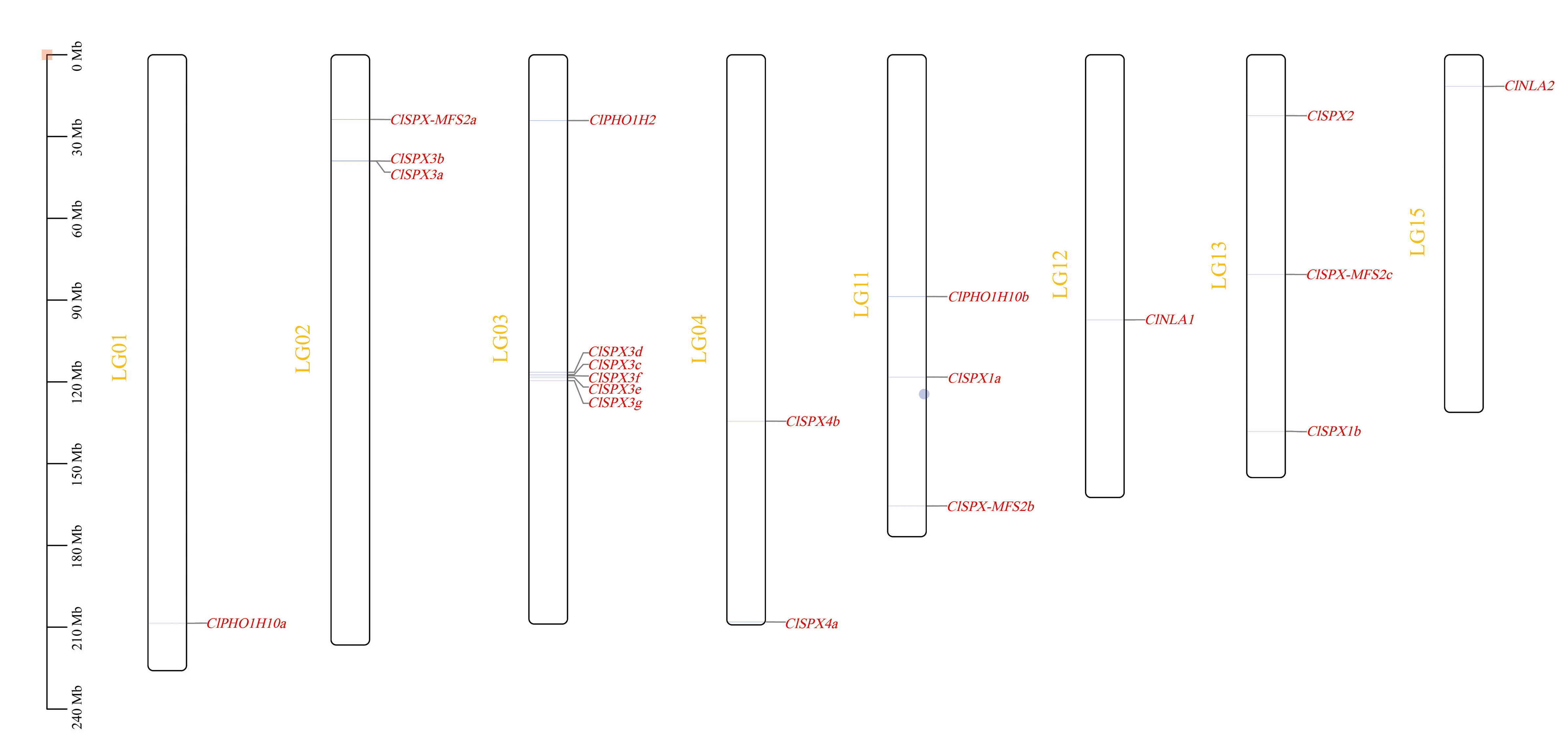

Supplement: Supplementary file 1 [file ijms-24-11552-s001.zip › Supplemental Figure S1.tif]

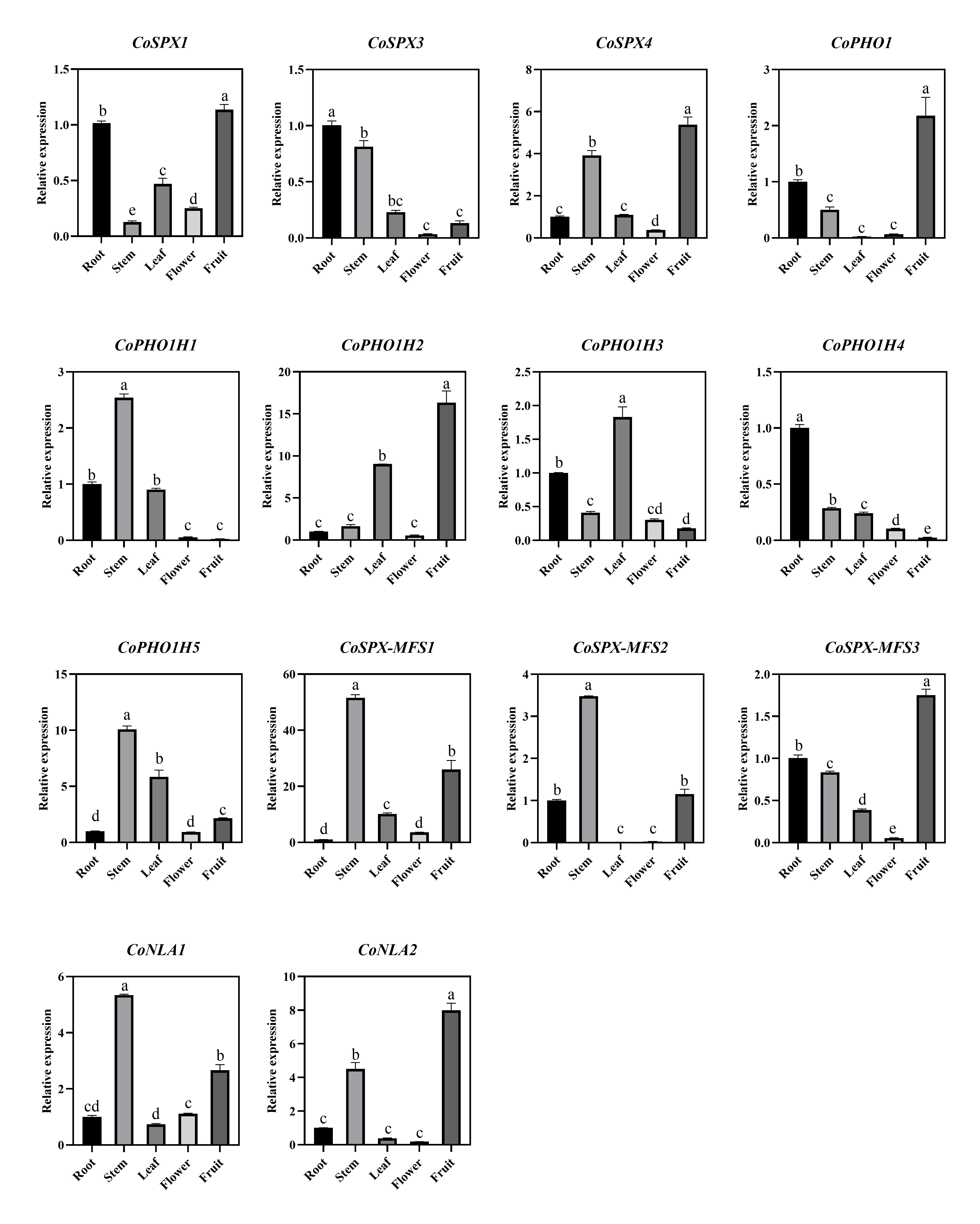

Supplement: Supplementary file 1 [file ijms-24-11552-s001.zip › Supplemental Figure S2.tif]

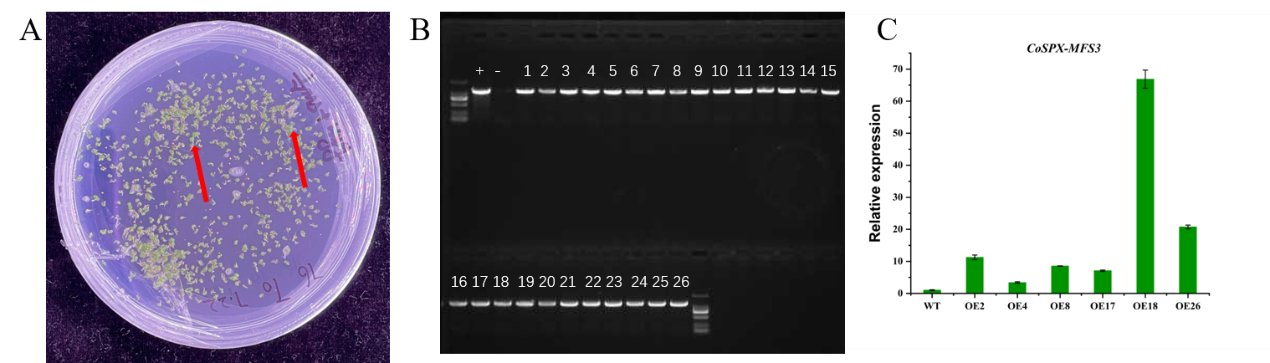

Supplement: Supplementary file 1 [file ijms-24-11552-s001.zip › Supplemental Figure S3 .tif]
